# Different abilities needed at home and school: The relation between executive function and adaptive behaviour in adolescents with Down syndrome

**DOI:** 10.1038/s41598-020-58409-5

**Published:** 2020-02-03

**Authors:** Camila Sabat, Paulina Arango, Marc J. Tassé, Marcela Tenorio

**Affiliations:** 1 School of Psychology, Universidad de los Andes, Monseñor Álvaro del Portillo 12455, Las Condes, Santiago, RM 7620111 Chile; 2 Nisonger Center, The Ohio State University, 1581 Dodd Drive, Columbus, OH 43210 USA

**Keywords:** Human behaviour, Disability

## Abstract

Studies have shown that executive function abilities are related and have predictive power over adaptive behaviour in both typical and atypical populations. This study examined the relationship between executive functioning and adaptive behaviour in adolescents with Down syndrome, as it has not been studied before in this population. We propose and test a model of how each core EF (i.e., working memory, inhibition, and flexibility) contributes to each domain of AB (i.e., conceptual, social, and practical). We found that parent reported Conceptual skills were related to working memory, while teacher reported Conceptual and Practical skills were related to inhibition and flexibility. We hypothesise that these findings are related to the different requirements and expectations of the home and school environments: the more predictable home environment requires the adolescent to rely on working memory for his everyday activities, while the changing and challenging school environment requires the inhibition common behaviours and to flexibly change actions to be successful.

## Introduction

Down syndrome (DS) is the most common chromosomal disorder, with a total mean prevalence (live, stillbirths and termination of pregnancies) of about 18.2 in 10,000 births^1^. In most cases, DS is caused by an extra copy of all or part of chromosome 21^2^, and it is considered one of the leading genetic causes of intellectual disability (ID)^3^, as cognitive functioning and adaptive behaviour (AB) seem to be somehow affected by the condition^2,4^.

Regarding cognitive functioning, executive function (EF) appears as a set of skills that have consistently been shown to be different in people with DS when compared to typically developing (TD) children and adults^4–6^. EF is a set of top-down skills used in the conscious control of attention, thoughts, and actions, when relying on our automatic processes would be unwise, insufficient, or impossible^7,8^. The three core EFs include working memory (i.e., the ability to hold information in mind and mentally work with it^9^), inhibition (i.e., the capacity to override automatic or impulsive responses, thoughts, or emotions, in order to act according to one’s goals and/or what is appropriate for the situation^7,10^), and cognitive flexibility (i.e., the ability that allows for change in perspectives, means to reach a goal, or the goal itself, in order to optimize resources and/or more effective use of the feedback from the environment^7,10^). These abilities form the foundation from which higher order cognitive processes, such as reasoning, problem solving, and planning are built. This multidimensional approach to EF has been supported by studies in children^11,12^ and adults^13^.

Studies that have explored EF in DS have reported that children and adolescents with DS have overall difficulties in EF, but with an age dependent profile of strengths and weaknesses across the different dimensions. Two studies with pre-school children with DS^6,14^ reported greater difficulties in working memory, planning, and inhibition, comparing with norms. Emotional control and flexibility were at level with their other cognitive abilities and with mental age matched peers. In school-aged children and adolescents with DS, working memory, planning, inhibition, and flexibility were reported to be areas of difficulty; while fluency and emotional control were found to be relative strengths^6,15^.

Overall, working memory has been consistently reported as an area of difficulty in people with DS^6,14–18^. Evidence has suggested that DS is associated with deficits in the phonological loop, particularly in storage and not in the rehearsal function. The functioning of the visuospatial sketchpad seems to be aligned with the general level of intellectual functioning, thus it does not appear impaired when considering mental age^17,19,20^. Studies have also consistently showed that, along with phonological loop problems, individuals with DS also show impairments in the central executive reflected in difficulties to perform working memory tasks that require increased control^19–21^. It is important to note that these difficulties are not associated with other problems commonly reported in people with DS, such as hearing impairment and/or articulation difficulties^17,20^.

Inhibition has been studied with mixed results in DS. Some studies with both children and adults have reported no differences in prepotent response inhibition among participants with DS and matched TD or controls with learning disabilities^22–24^, or only moderate difficulties^6^. On the other hand, several other studies have reported difficulties in verbal response inhibition when compared to matched participants with Williams syndrome, idiopathic ID, or TD^14,15,18,25,26^.

Regarding flexibility, findings have also been mixed. As stated before, Loveall and colleagues^6^ found flexibility as a strength in their pre-school DS group, and as a weakness in the school-age DS group. A couple of studies reported levels of flexibility that were comparable with cognitive ability levels^14^ or moderately impaired^18^. Several other studies with children, adolescents, and adults with DS have shown impairments on set-shifting tasks, when compared to norms, children and adolescents with Williams syndrome, or TD subjects matched for mental age^8,15,22,24,25,27,28^.

AB is another area of functioning that is usually impaired in people with DS^2,4^. AB corresponds to a set of learned skills necessary for everyday living and independence. They are commonly conceptualized as three different groups of abilities: (1) conceptual skills, including language and the understanding of time, money, and number concepts; (2) social skills, which involves interpersonal abilities, social problem solving, following rules and laws, etc.; and (3) practical skills, corresponding to personal care, occupational and safety capabilities, use of money and transportation, and following of schedules and routines^29^.

AB is an essential component in the diagnosis of ID, as important as intellectual functioning^30^. Furthermore, it is adaptive functioning that determines both the severity of the condition and the level of support required^31^. The general AB profile described in pre-school children with DS is characterized by relative strengths in the social and practical domains, and deficits in conceptual domain (i.e., language) and motor skills^32^. In adulthood, social skills remain a strength, while communication and practical skills appear as relative weaknesses^33^.

Studies with different populations have reported a relationship between EF and AB. For example, a strong association was reported between EF measures and the ability to work, drive, manage finances, and live independently in adults after acquired brain injury^34^. A study with children with heavy prenatal alcohol exposure and children with ADHD (without prenatal alcohol exposure), showed that both groups had lower scores on EF tasks and AB measures, than TD children^35^. When considering particular EF and AB domains, studies with typical and atypical populations have found that metacognitive processes (e.g., working memory, planning, problem solving) make the most significant contribution to all three domains of AB^36–38^. Studies have reported that conceptual and practical skills are better predicted by working memory in children with ASD^39,40^, inhibition in children with mild ID^41^, and flexibility in TD children^42^ and older adults^43^. These diverse findings could be related to the different typical and atypical populations analysed in the studies, and/or the different measures used. On the other hand, social skills have been consistently shown to be better predicted by flexibility across populations^39,40,42^.

One previous study has indirectly referred to the relation between EF and AB in people with DS. Adams and Oliver^44^ found no significant differences or declines in adaptive behaviour between groups of adults with DS with or without cognitive deterioration (including in EF) associate to dementia. This may point out that adaptive behaviour is not influenced by EF in adults with DS; although no information is available considering the childhood or adolescent period, where this abilities are developing.

Based on this, we have two main objectives:

1. To investigate the possibility of a relationship between EF and AB in adolescents with DS.
2. To propose and test a model of how each core EF (i.e., working memory, inhibition, and flexibility) contributes to each domain of AB (i.e., conceptual, social, and practical).

Regarding our first objective, we hypothesised that we would find a correlation between EF and AB, consistent with previous findings in other populations. To the extent of our knowledge, there are no studies that have explored something similar to our second objective. Since the research available is not consistent regarding which EF ability is more relevant to which AB domain, we developed our model based on previous research and on the theoretical understanding of these cognitive functions.

**Figure 1:**
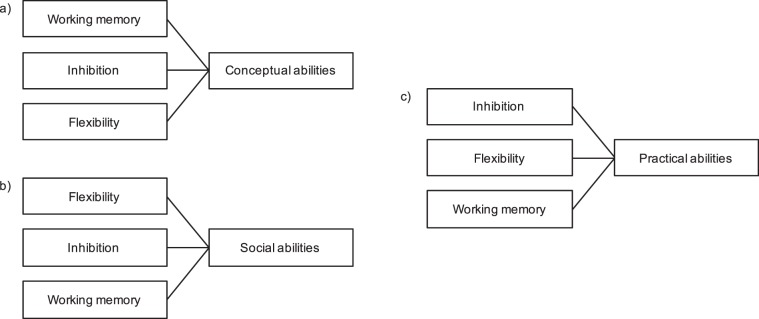
Proposed model of contribution of each core EF to every domain of adaptive behavior. (**a**) For conceptual abilities, we hypothesize working memory will be the main contributor, followed by inhibition, and then flexibility. (**b**) For social abilities, we hypothesize flexibility as the main contributor, followed by inhibition, and then working memory. (**c**) For practical abilities, we hypothesize that inhibition will be the first contributor, followed by flexibility, and working memory.

First, we hypothesised that working memory would be the best predictor of conceptual skills, as is critical for understanding written and spoken language, resolving mathematical problems, translating instructions into action plans, and several other abilities that are a key part of these skills^7^. We also hypothesised that inhibition would be the second best predictor, followed by flexibility, since controlling our attention, thoughts and behaviour, and the ability to shift between the best responses, would be beneficial in the learning process and use of conceptual skills (Fig. 1a).

Regarding the social domain, flexibility seems to be the core EF as the most likely factor to correlate with these adaptive skills^39,40,42^. Thus, we hypothesised that flexibility would be the best predictor of social skills. We believed it would be followed by inhibition and working memory, as these abilities allow a person to suppress behaviours that might damage the relationship with the other and bring information into mind about the people they are interacting with (Fig. 1b).

Finally, regarding the practical domain, we hypothesised that these skills would be better predicted by inhibition, followed by flexibility, and working memory. This hypothesis rests on the knowledge that these abilities allow a person to stop or avoid an activity that might be more interesting, in order to flexibly switch to another one that is more adaptive to the environment or the person’s needs. In the third place will be working memory, as an ability that can help to bring back to mind and use previous learned knowledge and experience about how to perform certain activities of daily living (Fig. 1c).

We believe this study will contribute to our understanding of both EF and AB, and how they are related to each other, particularly in adolescents with DS. Furthermore, our proposed three models of significance of core EFs to AB domains can give significant information that could help understand certain difficulties that some adolescents with DS might deal with while learning AB skills and can subsequently guide our interventions.

## Method

### Participants

Participants were recruited with the assistance of DS organizations in Santiago, Chile. The inclusion criteria were: (1) to have a confirmed diagnosis of DS by karyotype, (2) chronological age between 12 and 17 years old, and (3) have the ability to communicate through oral language. For our study, we also excluded participants who presented with a comorbid psychiatric disorder and/or other neurodevelopmental disorder (e.g., ASD) diagnosed by a professional. Participants were also excluded from the sample if they failed to complete all required testing. Thus, from the original sample of 56 adolescents, we excluded three participants due to the presence of comorbid ASD, and 17 participants were excluded from the sample due to incomplete or missing testing data. Our final sample consisted of 36 adolescents (6 females and 30 males) with a mean chronological age of 14.44 years (*SD* = 1.30). More descriptive information can be found in Tables 1 and 2 (IQ scores).

**Table 1:** Sample distribution by type of education and socioeconomic status.

	n	%
**Type of education**		
Regular^a^	18	50.00
Special^b^	7	19.40
NGO^c^	11	30.60
**Socioeconomic status**^**d**^		
Low^e^	6	16.70
Medium^f^	4	11.10
High^g^	26	72.20

*Notes:* ^a^General or mainstream schools with inclusion programs.

^b^Special schools for students with disabilities.

^c^Non-governmental organizations that offer specialized intervention programs to children and adolescents with Down syndrome.

^d^As measured by monthly family income.

^e^Less than $178,334 to $558,069 Chilean pesos.

^f^$558,070 to $2,439,954 Chilean pesos.

^g^More than $2,439,955 Chilean pesos.

**Table 2:** Group scores on IQ, working memory, inhibition, and flexibility measures.

Ability	Measure	*M*	*SD*	Range (min-max)
Intellectual functioning	WISC-IIIv.ch./WAIS-IV^a^			
	Verbal IQ^b,e^	48.36	5.94	40–67
	Performance IQ^c,e^	46.28	3.79	40–59
	Total IQ^d,e^	41.17	2.41	40–53
Working memory	Digit span^a^			
	Forward^f^	1.28	1.23	0–4
	Backward^f^	1.00	1.24	0–4
	Total^f^	2.28	2.26	0–7
Inhibition	HF^a^			
	Congruent items^f^	14.86	6.60	5–28
	Incongruent items^f^	16.17	8.93	0–29
Flexibility	WCST perseverative responses^a^			
	Raw score^g^	41.94	17.12	10–62
	Standard score^h^	5.15	3.33	−0.21–12.23

*Notes:* ^a^*n* = 36.

^b^WISC-III or WAIS-IV verbal IQ. ^c^WISC-III performance IQ or WAIS-IV perceptual reasoning index. ^d^WISC-III or WAIS-IV full scale IQ. ^e^Standard IQ scores (*M* = 100, *SD* = 15). ^f^Raw scores (total correct). ^g^Total perseverative responses. ^h^*z*-scores (*M* = 0, *SD* = 1).

### Procedure

After the initial contact, the parents and participants were invited to an in-person meeting where the objectives and procedures of the study and intervention were explained to them. Informed consent and assent forms were reviewed, accepted, and signed by the parents and adolescents who agreed to participate.

Participants were assessed following a standard procedure including either the Wechsler Intelligence Scale for Children-3^rd^ edition, Chilean adaptation and standardization (WISC-IIIv.ch.)^45,46^ or the Wechsler Adult Intelligence Scale-4^th^ edition, Chilean adaptation and standardization (WAIS-IV)^47,48^, depending on their chronological age (16 years old and younger were assessed with the WISC-IIIv.ch., while 17 years old or older were assessed with the WAIS-IV); the Wisconsin Card Sorting Test (WCST)^49^; and the Hearts and Flowers task (HF)^50^. The parents and teachers of the participants were asked to independently complete the Adaptive Behaviour Assessment System-II Spanish adaptation and standardization (ABAS-II)^51,52^.

This study was conducted in accordance with the ethical standards presented in the Declaration of Helsinki. Ethical approval was obtained from the Institutional Review Board for Ethical Research at the Universidad de los Andes (Chile).

### Measures

We measured working memory using the sum of the total correct spans from the forward and backward digit span tasks of the WISC-IIIv.ch^45,46^. or the WAIS-IV^47^. Digit span tasks have been thoroughly used as measures of immediate verbal recall, attentional capacity, and working memory, in both neuropsychological clinical assessments and research^53^. This task usually consists of two modalities: forward and backward (the WAIS-IV includes sequencing, which was not used for purposes of this study). It has been proposed that digit span forwards requires the involvement of the phonological loop, while digit span backwards should also engage the central executive^9,53,54^. Reliability has been supported for the Digit Span task on both the WISC-IIIv.ch. (α = 0.65)^45^ and the WAIS-IV (α = 0.88)^55^.

Inhibition was assessed using the total correct incongruent responses of the HF task^50^. Previously known as The Dots test, this task was developed in order to address both working memory and inhibition^50,56,57^. There are three blocks on this test: first, the congruent block, were participants tap the screen on the same side the stimulus (a heart) appears; second, the incongruent block, were participants tap the opposite side on which the stimulus (a flower) appears; and third, the mixed block, were participants have to switch between those two rules, depending on the stimulus showed. A study by Wright and Diamond^56^, showed that incongruent responses are more difficult because they add an inhibitory demand.

Finally, flexibility was measured using the total number of perseverative responses of the WCST^49^. The WCST evaluates abstract concepts, set shifting and maintenance, and feedback use, and is one of the most widely used tests for executive function^58–60^. In this task, participants have to sort cards following a specific principle, but they are not told what the principle is or when it changes, having to infer that from the feedback of the examiner^49^. Perseverative responses occur when the person insists on responding according to a category that is not correct. Reliability and validity of the WCST have been extensively supported^49,58^.

The three domain scores (conceptual, social, and practical) of ABAS-II^51,52^ were used to measure AB, our dependent variable. This scale assesses a wide range of skills necessary for personal and social competence in daily activities; and it yields standard scores in the three AB domains. All these scores are expressed on a standard metric (*M* = 100, *SD* = 15). The ABAS-II is completed directly by a respondent, typically the assessed person’s parent and/or teacher^51^. Reliability and validity have previously been established^51^. We decided to measure AB with a Spanish adaptation normed on a comparable Spanish population, since there are no standardized Chilean tests for the assessment of this ability.

### Statistical analyses

Data were analysed using SPSS Statistics 25^61^. To test our first research objective, we conducted linear correlation analyses using Pearson *r* between the EF and AB measures. We conducted multiple regression analyses using the enter method, to test the contribution of each core EFs to AB domains. In each regression we tested three models: the first model only includes the measure added on the first step, the second model includes the measures added on the first and second steps, and the third model includes the measures added on the first, second, and third steps. For the Conceptual domain, we added the measure of working memory on the first step, the measure of inhibition on the second step, and the measure of flexibility on the third step. For the Social domain, we included flexibility in the first step, inhibition in the second step, and working memory in the third. Finally, for the Practical domain, we added inhibition in the first step, flexibility in the second step, and working memory in the third step.

## Results

Regarding the performance on EF, the mean scores and standard deviations for each EF measure (i.e., digit span, HF, and WCST) can be found in Table 2. Digit span showed a floor effect, concordant with the difficulties reported in this area in DS^6,14–18^. Participants were able to correctly repeat, on average, 1.28 of the forward and 1.00 of the backwards digit spans presented (2.28 in total). No significant differences were found between the average correct congruent and incongruent items of the HF task (*p* > 0.05). On average, the participants obtained perseverative responses scores that were 5.15 standard deviations above the norm in the WCST.

The mean scores of each AB domain (i.e., Conceptual domain, Social domain, and Practical domain) as reported by parents and teachers can be found in Table 3. Significative differences were found only in the Practical domain, in favour of teacher report.

**Table 3:** Group adaptive behavior scores.

	Parents (*n* = 36)			Teachers (*n* = 35)				*p*-value
	*Mean*	*SD*	Range (min-max)	*Mean*	*SD*	Range (min-max)	*t*	
Conceptual domain^a^	65.08	13.64	54–112	67.11	11.10	51–92	−0.84	0.407
Social domain^a^	81.86	18.89	51–128	86.14	12.20	56–112	−1.31	0.198
Practical domain^a^	69.03	12.79	51–103	76.17	12.05	51–102	−2.62	0.013

*Notes:* ^a^in standard scores (*M* = 100, *SD* = 15).

Considering our first objective, the results showed significant moderate positive correlations between the Conceptual domain as reported by parents with the digit span total raw score. No other AB domain reported by parents had significant correlations with the EF measures. Regarding teacher report, there were significant moderate positive correlations between the Conceptual domain and the digit span total raw score and the HF incongruent responses, and a negative moderate correlation with the total perseverative responses of the WCST. The Practical domain had moderate positive correlations with the HF incongruent responses, and moderate negative correlation with the total perseverative responses of the WCST. All correlations can be seen in Table 4.

**Table 4:** Correlations between executive functions and adaptive behavior.

	Digit span^a,c^		HF incongruent responses^a,c^		WCST perseverative responses^a,d^	
	*r*	*p*	*r*	*p*	*r*	*p*
**ABAS-II Parents**^**a**^						
Conceptual domain	0.40	0.015*	0.30	0.079	−0.28	0.130
Social domain	0.19	0.274	0.16	0.347	−0.17	0.336
Practical domain	−0.01	0.940	0.25	0.144	0.078	0.651
**ABAS-II Teachers**^**b**^						
Conceptual domain	0.35	0.041*	0.53	0.001*	−0.52	0.001*
Social domain	−0.02	0.932	0.27	0.123	−0.30	0.085
Practical domain	0.21	0.227	0.42	0.011*	−0.41	0.014*

*Notes:* ^a^*n* = 36.

^b^*n* = 35. ^c^Raw scores (total correct). ^d^Raw score (total responses). *Significant correlations.

**Figure 2:**
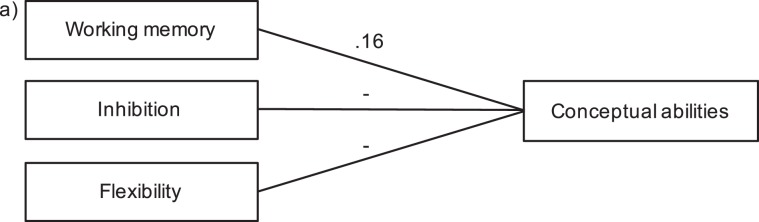
Results of the regression analyses of the Conceptual domain as reported by parents. Working memory (digit span) was added on the first step; working memory plus inhibition (HF) on the second step; and working memory plus inhibition plus flexibility (WCST) on the third step. Results show that working memory predicted 16% of the variance, *β* = 0.40, p = 0.015.

To tests our second objective, we started with the Conceptual domain as reported by parents. The results showed that all three models were significant (first model: working memory; *R*^2^ = 0.16, *F* (1,34) = 6.55, *p* = 0.015; second model: working memory + inhibition: *R*^2^ = 0.22, *F* (2,33) = 4.72, *p* = 0.016; third model: working memory + inhibition + flexibility: *R*^2^ = 0.23, *F* (3,32) = 3.14, *p* = 0.039), though only the first model had a significant *R* square change and digit span was the only significant predictor of the Conceptual domain (both *p* = 0.015) as can be seen in Fig. 2. Thus, only the first model was considered relevant.

**Figure 3:**
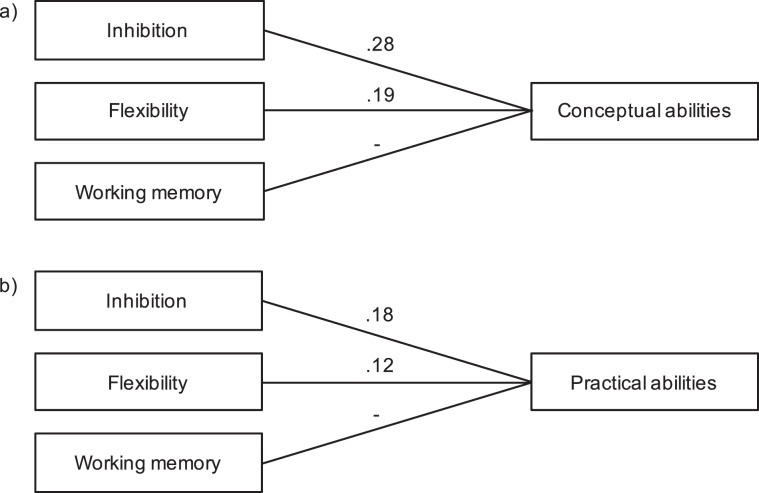
Results of the regression analyses of the Conceptual and Practical domains as reported by teachers. For both conceptual and practical skills, inhibition (HF) was added on the first step; inhibition plus flexibility (WCST) on the second step; and inhibition plus flexibility plus working memory (digit span) on the third step. (**a**) For conceptual abilities, inhibition predicted 28% of the variance, *β* = 0.45, p = 0.002; while flexibility explained the other 19%, *β* = −0.45, p = 0.002. (**b**) For practical abilities, inhibition predicted 18% of the variance, *β* = 0.36, p = 0.022; while flexibility explained the other 12%, *β* = −0.35, p = 0.026.

Regarding the Conceptual domain as reported by teachers, we found that the three models were significant; however, both the HF and WCST measures explained a higher amount of variance than digit span, and the working memory measure was not a significant predictor of the Conceptual domain. Based on this, we decided to repeat the regression, changing the order of the factors. We first added the measure of inhibition, followed by flexibility, and finally working memory (Fig. 3a). With this adjustment, all three models reached significance (first model: inhibition; *R*^2^ = 0.28, *F* (1,33) = 12.62, *p* = 0.001; second model: inhibition + flexibility: *R*^2^ = 0.47, *F* (2,32) = 14.15, *p* < 0.001; third model: inhibition + flexibility + working memory: *R*^2^ = 0.49, *F* (3,31) = 9.72, *p* < 0.001); although, the *R* square change of the third model was not significant (*p* > 0.05) and only inhibition and flexibility were significant predictors of the Conceptual domain (Fig. 3a). Thus, we only considered the first two models.

For the social domain, no model reached significance for either parent or teacher reports (all *p* > 0.05).

No model reached significance when considering the Practical domain skills reported by parents (all *p* > 0.05). When using the teacher report, as with previous results, all three models reached significance (first model: inhibition; *R*^2^λ0.18, *F* (1,33) = 7.17, *p* = 0.011; second model: inhibition + flexibility: *R*^2^ = 0.30, *F* (2,32) = 6.79, *p* = 0.003; third model: inhibition + flexibility + working memory: *R*^2^ = 0.30, *F* (3,31) = 4.40, *p* = 0.011); although, again we considered only the explained variance of the measures of inhibition and flexibility, since the *R* square change of the third model was not significant and working memory was not a significant predictor (all *p* > 0.05; Fig. 3b).

## Discussion

Following our first objective, we hypothesised a relationship between EF and AB in adolescents with DS. Indeed, we found moderate correlations between conceptual skills (as reported by parents and teachers) with working memory, as well as conceptual and practical skills (as reported by teachers) with inhibition and flexibility. There were significant differences in the Practical domain as reported by parents and teachers. These differences have been addressed in previous research. Montero-Zenteno and Fernández-Pinto^51^ referred good levels of agreement between different informants on the ABAS-II, but modest correlation coefficients between parent and teacher respondents. This has been explained by the fact that the individuals being assessed often perform differently across different environments with differing expectations. Accordingly, studies with the Vineland Adaptive Behaviour Scale also found great general agreement between raters, but higher overall scores reported by teachers than parents^62,63^. These findings are key to our study, as they help explain not only why parents and teachers report different levels of AB skills, but also why we found that the three core EF were differently related across AB domains, depending on whether it was reported by parents or teachers.

Accordingly, we found a stronger correlation between EF and teacher reported AB, than EF and parent reported AB. One possible explanation is that EFs are required during novel activities and situations^7^, thus they are a more needed cognitive ability to be accessed during school activities, which are characterized by continuous learning of new concepts and skills, compared to the more predictable and routine adaptive skills expected in a home setting.

To further explore the relationship between core EFs and AB domains, we developed a second hypothesis, stablishing that each core EF will have a different weight for each AB domain. Regarding conceptual adaptive skills, although we found a correlation with working memory, this cognitive ability by itself was significantly predictive of only the AB Conceptual domain score as reported by parents. Neither inhibition nor flexibility was significant. On the other hand, when exploring conceptual adaptive skills as reported by teachers, we found that inhibition and flexibility were significant predictors, while working memory did not reach significance. Thus, our hypothesis was only partially confirmed by our findings when considering parent reported AB and was rejected when considering teacher reported AB. Nevertheless, our results seem to be consistent with previously reported findings that metacognitive abilities (which include working memory along other EF skills) predicted conceptual skills^36,37,39,40^, and other studies that found inhibition^41^ and flexibility^42^ to be significantly correlated with conceptual skills. Interestingly, all studies that found metacognitive abilities as better predictors of conceptual skills used caregiver (mostly parents) reports of everyday executive functions^36,37,39,40^, while the studies that highlighted the importance other core EF: inhibition^41^ and flexibility^42^, used tests that measured EF directly in the participant. Based on this, we hypothesised that working memory would play a more significant role in the execution of activities related to conceptual skills at home, a more predictable setting where the need to learn and perform new skills occurs less frequently than in a school setting. The systematic and constant learning of new abilities and concepts required in the classroom implies the need for a different set of EFs: inhibition and flexibility, that is, the abilities to inhibit a common response and look for and use a novel one^7^.

No EF measure either correlated or had predictive power over the Social domain of AB, as reported by parents or teachers, rejecting our hypothesis. Our findings also differ from previously published studies that reported flexibility as a predictor of social skills^39,40,42^. It is important to note that the previous studies involved samples of children with ASD and TD. As stated before, research (including this study) has consistently showed that social skills are a relative strength in people with DS, compared to other adaptive and intellectual skills^32,33^, while EF has been reported as a difficulty in people with DS^16,27,64^. Based on this, our hypothesis is that people with DS might tap other abilities to develop their social skills, including understanding and successfully resolving social situations. Another possible explanation to our findings is that, since social skills tend to be a relative strength for people with DS, they receive more attention and encouragement from parents and other adults for these skills, thus, by the time the child with DS becomes an adolescent, EFs will not be as relevant as other skills or factors, for example the use of contextual cues, to support further developmental of social skills. More research is needed to test these hypotheses.

As with conceptual skills, we found a difference in the predictive power of our model between parents’ and teachers’ reported practical skills. No core EF were significant predictors of parent reported practical skills; however, we found inhibition and flexibility to be a significant predictor of teacher reported practical skills, while working memory did not reach significance. Thus, again our results partially support our hypothesis. As previously, we believe that our findings could be explained in part by the different environments in which the adolescents with DS perform their adaptive behaviour. On one hand, the home environment might be more predictable, and they might have more support from their parents and siblings, who can help the adolescents with everyday chores and activities. Thus, they might not have the need to access their EFs to perform these skills. On the other hand, the school environment changes rapidly, there is less individual support from adults or peers, and more autonomy is progressively required on the part of the student. In this scenario, the ability to inhibit and think before acting and to be able to flexibly adapt one’s behaviour to the environment becomes essential.

In conclusion, we found that for our group of adolescents working memory was a significant predictor only for parent reported conceptual skills, and inhibition and flexibility were significant predictors of teacher reported conceptual and practical skills. We postulate that the differences in the relation between EF and AB may be associated to the different demands across the home and school environments. In the home environment, since activities and expectations are different, it is possible that the individual with DS follows predictable and repetitive patterns of behaviour and gets more support from others, in order to accomplish everyday tasks, lessening the need to rely on EF. The person with DS’s relative strength in visuospatial working memory^17,19,20^ might explain why working memory is significant for parent reported conceptual skills but not teachers reported conceptual skills, since they might be able to rely more on visuo-spatial information in the home environment than in the school environment, where most instructions and activities have a stronger verbal component. As previously stated, inhibition and flexibility become more important in the school setting, where change is more frequent and requirements increase in difficulty. Following this, it is important to also consider that both parents and teachers have different expectations and demands regarding AB, and consequently might offer more or less support and promote more or less autonomy in the adolescents.

This research was a first approach to the study of EFs and AB in adolescents with DS. Although we found correlations between core EFs and AB domains, none of our hypotheses regarding our three models of contribution of core EFs to AB domains were completely supported. Our model was based on previous research involving samples of typically developing and other disability groups, none of which were people with DS, hence, it is possible that the relation between EFs and AB is different in this population. Although more research is needed to further our understanding of the development of EFs in people with DS, this study offers evidence that could be useful for future applied research regarding interventions and supports in AB.

There are several limitations in our study. First, we had a small sample size. Second, we decided to use raw scores when possible. This means that age differences in EF scores were not considered. Since the age range is small and whether their scores were or not on the average for age was not a factor considered, we used the raw scores in order to be able to preserve as much variance as possible. Due to the characteristics of the tests used to assess our constructs, however, only the EF measures yielded raw scores, the AB measures yielded standard scores. It is possible that we lost some of the variance for our sample in their AB measures, affecting our results. Third, our sample consisted of students that are integrated into regular schools, attending special schools, or attending a non-Government Organization that offer special programs for people with DS. Each of these settings has different requirements and expectations for their students. Consequently, EFs could be differently related to AB domains depending on the type of educational setting in which the adolescent is receiving their education. Given our sample sizes, we were unable to test this hypothesis. The ABAS-II scores should also be interpreted with caution since the norms used are based on a Spanish population and not a Chilean population. However, these norms have been used in previous studies of AB in people with DS in Chile, with good results^65^. Finally, there is evidence that EFs can be affected by socioeconomic status^66^. Most of our sample consisted of adolescents with DS that come from families with high SES, which could have affected our results. Future studies should address these limitations.

Positive contributions of our study include the comprehensive assessment of EFs and AB of a group of adolescents with DS. We also proposed and tested a novel model of significance of each core EF to the three AB domains. Although we did not confirm our hypotheses fully, we offer interesting findings regarding the EF abilities of these adolescents and how they relate to their AB.

The datasets generated during and/or analysed during the current study are available from the corresponding author on reasonable request.
